# Viral Shedding in Patients Infected with Pandemic Influenza A (H1N1) Virus in Kenya, 2009

**DOI:** 10.1371/journal.pone.0020320

**Published:** 2011-06-10

**Authors:** Lilian W. Waiboci, Emmaculate Lebo, John M. Williamson, William Mwiti, Gilbert K. Kikwai, Henry Njuguna, Beatrice Olack, Robert F. Breiman, M. Kariuki Njenga, Mark A. Katz

**Affiliations:** 1 US Centers for Disease Control and Prevention-Kenya, Nairobi, Kenya; 2 Kenya Medical Research Institute/US Centers for Disease Control and Prevention-Kenya, Nairobi, Kenya; 3 Influenza Division, National Center for Immunization and Respiratory Diseases (NCIRD), Centers for Disease Control and Prevention, Atlanta, Georgia, United States of America; University of Texas Medical Branch, United States of America

## Abstract

**Background:**

Understanding shedding patterns of 2009 pandemic influenza A (H1N1) (pH1N1) can inform recommendations about infection control measures. We evaluated the duration of pH1N1 virus shedding in patients in Nairobi, Kenya.

**Methods:**

Nasopharyngeal (NP) and oropharyngeal (OP) specimens were collected from consenting laboratory-confirmed pH1N1 cases every 2 days during October 14–November 25, 2009, and tested at the Centers for Diseases Control and Prevention-Kenya by real time reverse transcriptase polymerase chain reaction (rRT-PCR). A subset of rRT-PCR-positive samples was cultured.

**Results:**

Of 285 NP/OP specimens from patients with acute respiratory illness, 140 (49%) tested positive for pH1N1 by rRT-PCR; 106 (76%) patients consented and were enrolled. The median age was 6 years (Range: 4 months–41 years); only two patients, both asthmatic, received oseltamivir. The median duration of pH1N1 detection after illness onset was 8 days (95% CI: 7–10 days) for rRT-PCR and 3 days (Range: 0–13 days) for viral isolation. Viable pH1N1 virus was isolated from 132/162 (81%) of rRT-PCR-positive specimens, which included 118/125 (94%) rRT-PCR-positive specimens collected on day 0–7 after symptoms onset. Viral RNA was detectable in 18 (17%) and virus isolated in 7/18 (39%) of specimens collected from patients after all their symptoms had resolved.

**Conclusions:**

In this cohort, pH1N1 was detected by rRT-PCR for a median of 8 days. There was a strong correlation between rRT-PCR results and virus isolation in the first week of illness. In some patients, pH1N1 virus was detectable after all their symptoms had resolved.

## Introduction

The 2009 pandemic influenza A (H1N1) (pH1N1) virus has been circulating worldwide since the initial cases were detected in the United States in April 2009 [1]. In order to control the spread of infection, public health agencies including the World Health Organization (WHO) and the United States Centers for Disease Control and Prevention (CDC) made recommendations on the duration of isolation of pH1N1 cases. The recommendations were based on previous knowledge about duration of shedding of seasonal influenza viruses [2], [3], [4]. Most patients infected with seasonal influenza shed the virus for 5–7 days. However, children have been shown to shed the virus for up to 21 days and severely immunocompromised individuals up to several months [2], [5]. Following the onset of the influenza pandemic in 2009, WHO recommended that control precautions such as self-isolation should be practiced for 7 days from symptom onset or until all symptoms resolve [6].

Much of the information on pH1N1 virus shedding is from studies conducted in North America, Asia, and Europe [7], [8], [9], [10], [11], [12]. In many of these studies, real time reverse transcriptase polymerase chain reaction (rRT-PCR) positive results were used to identity positive cases and determine the duration of virus shedding. A study in Hong Kong found that pH1N1 virus RNA was shed from the respiratory tract for up to 8 days (median = 4 days) after symptom onset, [10]. Studies carried out in China and Germany showed that pH1N1-infected patients shed the virus for a mean of 6 days (Range: 1–17) and 6.6 days (Standard deviation: 2.6), respectively [10], [11]. In addition, a case report from the United States reported that pH1N1 virus was detected in respiratory samples 5 to 6 weeks after initial diagnosis in 2 severely immunocompromised patients on chemotherapy; these patients were on oseltamivir treatment and developed resistance to the drug [9].

The first case of pH1N1 in Kenya was detected in June 2009 [13]. At that time, the Kenya Ministry of Public Health and Sanitation followed WHO guidelines and recommended 7 days of self-isolation for all pH1N1 patients. In order to assist in providing evidence-based recommendations for infection control, we tested serial specimens from laboratory-confirmed pH1N1 patients attending a clinic in Nairobi to determine the duration of viral shedding, determine the correlation between rRT-PCR-positive results and viral isolation, and evaluate clinical signs and symptoms associated with shedding.

## Materials and Methods

### Ethics Statement

This study was approved by both the Institutional Review Board of CDC-Atlanta and the Ethical Review Committee of the Kenya Medical Research Institute (KEMRI).

### Consent

Informed written consent was obtained from all participants. For children, written consent was obtained from parents or guardians.

### Setting and Study Design

We conducted the study in a large informal urban settlement in Nairobi, in an existing population-based surveillance system that has been previously described [14]. Consenting participants who presented to a field clinic, known as Tabitha Clinic, within the surveillance site with signs and symptoms consistent with influenza-like illness (ILI) or severe acute respiratory illness (SARI) had oropharyngeal (OP) and nasopharyngeal (NP) specimens taken. The specimens were tested for influenza by rRT-PCR at the KEMRI/CDC-K laboratory in Nairobi.

An ILI case was defined as a patient with fever ≥38°C and cough or sore throat for all ages. For SARI, the definitions varied by age group. In children <5 years old, SARI was defined as cough or difficulty breathing along with a danger sign; unable to drink or breast feed, lethargic or unconscious, vomiting everything, convulsions, nasal flaring, grunts, chest indrawing, stridor in a calm child or oxygen saturation ≤90%. In people ≥5 years, SARI was defined as fever ≥38°C plus cough or shortness of breath or chest pain or oxygen saturation ≤90%.

Patients who came to Tabitha Clinic between October 14, 2009, and November 25, 2009, who had ILI or SARI and NP/OP specimens that tested positive for pH1N1 were recruited for the study. Patients who were positive for pH1N1 were contacted at their homes by field workers and requested to return to the clinic for follow up every 2 days. During the initial visit and subsequent visits, a trained clinician recorded signs and symptoms and collected both NP and OP specimens. Patients who had 2 consecutive rRT-PCR negative specimens were released from the study. A patient was considered to have pH1N1 RNA if a specimen collected on that day was positive for pH1N1 by rRT-PCR. Patients were not followed over the weekend.

### Specimen collection and laboratory testing

Specimens were obtained according to the following procedure: for OP specimens, a sterile nylon-tipped plastic-shafted OP swab touched the back of the oropharyngeal mucosal membrane for 3–5 seconds and then was placed into a cryovial containing 1 mL of viral transport media (VTM). VTM was prepared at the KEMRI/ CDC-K laboratory using the standard WHO protocol that includes bovine serum albumin and veal infusion broth supplemented with amphotericin B (www.who.int/csr/resources/publications/surveillance/Annex8.pdf). Freshly prepared refrigerated VTM was used for up to 3 months. For NP specimens, a polyester-tipped flexible aluminum-shafted NP swab was inserted into the nose to the nasopharynx, where it was rotated 180 degrees and left in place for 3–5 seconds. The NP swab was inserted into the cryovial containing the OP swab from the same patient. The specimens were labeled and transported at 4°C to the KEMRI/CDC-K laboratory where they were tested for influenza A and pH1N1 using rRT-PCR within 24 hours.

The rRT-PCR testing was performed using the CDC pH1N1 testing protocol [15]. Briefly, total RNA was extracted from 100 µL aliquots of each specimen using QIAamp viral RNA minikit (Qiagen Inc., GmbH, Germany) according to the manufacturer's instructions. One step rRT-PCR was carried out using the AgPath kit (Applied Biosystems, California, USA). For influenza A detection, the primers used were 5′GAC CRA TCC TGT CAC CTC TGA C as the forward, and 5′ TG CAG TCC TCG CTC ACT GGG CAC G as the reverse. The influenza A detection probe was 5′ TGC AGT CCT CGC TCA CTG GGC ACG. For pH1N1, we used the 5′GTG CTA TAA ACA CCA GCC TYC CA as the forward primer, 5′ CGG GAT ATT CCT TAA TCC TGT RGC as the reverse primer, and 5′CA GAA TAT ACA TCC RGT CAC AAT TGG ARA A as the probe. Specimens were also tested for seasonal influenza A H1 and H3. Following the reverse transcription step, a typical 45 cycle PCR reaction was run and fluorescence was read at the annealing/extension step. Appropriate negative and positive control specimens were run alongside each reaction. The results were recorded as cross-over threshold (C_T_) values. Any influenza A C_T_ value <40.0 was recorded as positive; C_T_ value 40.0–44 were considered indeterminate, and those without a C_T_ reading were recorded as negative. A specimen was considered to be pH1N1 positive if both the influenza A and the pH1N1 C_T_ values were <40.0 as described in the CDC protocol of real time RT-PCR for influenza A(H1N1) [16].

Later, in order to evaluate the concordance between rRT-PCR testing and viral culture for pH1N1, influenza virus isolation in Mardin-Darby Canine Kidney (MDCK) cells was attempted for the first rRT-PCR-positive specimen, the last rRT-PCR-positive specimen, and the first rRT-PCR-negative specimen from each enrolled patient. Confluent monolayers of MDCK cell line growing in T25 cell culture flasks were used. Media were removed from the flasks. Each flask was inoculated with 100 µL of specimen and inoculum was allowed to adsorb on the cells for 30 min at 37°C. Following adsorption, 6 mL of Viral Growth Medium [DMEM supplemented with HEPES buffer, bovine serum albumin, L-Glutamine, trypsin, and antibiotic-antimycotic (Sigma-Aldrich, Inc., St. Louis, MO, USA)] was added and cultures were observed daily for cytopathic effect (CPE) for up to 6 days, at which point all the cultures were harvested. Cultures that showed observable CPE by microscopy were subjected to hemagglutination assay using guinea pig red blood cells. Supernatants from cultures that showed no CPE were subjected to a second passage, following which cultures with observable CPE were subjected to hemagglutination assay. Cultures that had no observable CPE after the second passage were considered negative by viral isolation.

### Data collection and statistical methods

Data were collected in three ways. First, information related to the patient's initial visit was recorded on computers by clinicians at Tabitha Clinic. Second, during the first follow-up clinic visit and subsequent visits, information about signs and symptoms was recorded on paper questionnaires and entered into a Microsoft Access database. The signs and symptoms recorded were fever, temperature ≥38°C, cough, sneezing, runny nose, vomiting, diarrhea, chest pain, and difficulty breathing for all patients. In addition, the following symptoms were recorded for patients ≥5 years; earache, sore throat, headache, chills, muscle pain, and abdominal pain. Third, laboratory data were recorded into Freezerworks software (Dataworks Development, Inc.). Data were combined into a central database for analysis.

We considered the date of onset of illness to be the date on which the patient reported the first symptom associated with the illness, according to the history taken during the patient's initial clinic visit. For possible initial symptoms, we considered fever, cough, and difficulty breathing for patients <5 years old. In patients ≥5 years old, these symptoms and sore throat were considered as possible initial symptoms. Patients were considered to have no detectable virus RNA once they had two consecutive specimens that tested negative for pH1N1 by rRT-PCR. We defined the last day of virus detection as the midpoint between the dates of collection of the last rRT-PCR pH1N1-positive specimen and the first rRT-PCR pH1N1-negative specimen. We were unable to follow all patients until they had two negative rRT-PCR tests because some did not return to the clinic for the necessary follow-up visits. The time to cessation of pH1N1 virus detection for these patients was right censored at their last pH1N1 rRT-PCR-positive test date. Thus, the duration of pH1N1 virus detection for these patients was only known to occur after their drop out time and was treated accordingly in the survival analysis below.

Comparisons of duration of pH1N1 virus detection between groups, described by the median and mean, were conducted using the log-rank test. A multivariable Cox regression model was used to determine the association between gender and age with duration of pH1N1 virus detection. A Kaplan-Meier plot was used to determine duration of pH1N1 virus detection. Odds ratios were calculated to determine the odds of isolating pH1N1 virus from rRT-PCR-positive specimens collected over time. All analyses were conducted with SAS version 9.1. Findings were considered statistically significant if the resulting p-value was <0.05.

## Results

**Figure 1:**
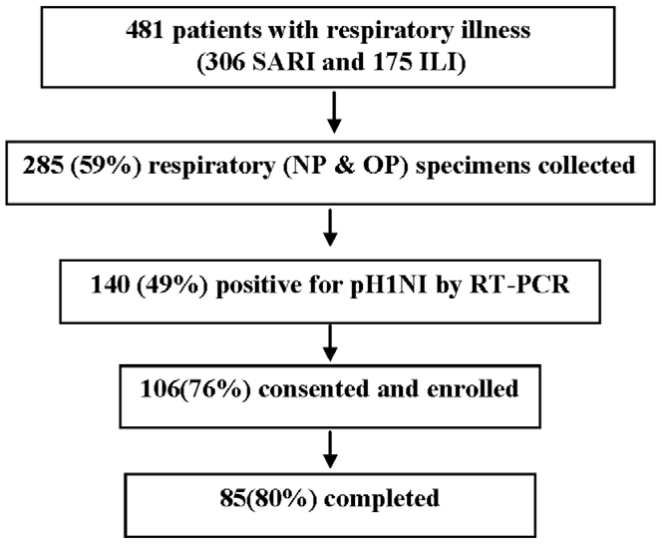
Flow chart outlining enrollment in the pH1N1 viral shedding study undertaken in Kenya from Oct 14–Nov 25, 2009.

A total of 481 patients with respiratory illness were seen at Tabitha Clinic between October 14, 2009 and November 25, 2009; 175 (36%) had ILI and 306 (64%) had SARI. Specimens were collected from 285 (59%) of these patients, and 140 (49%) of these were positive for pH1N1 by rRT-PCR. Of the 140 pH1N1-positive patients, 106 (76%) consented to participate in the study; 85 (80%) completed the study (Figure 1). Data from all the 106 patients who enrolled in the study were included in the analysis.

The median age of the study population was 6 years (Range: 4 months–41 years); 60 (57%) were females (Table 1). The mean duration between symptom onset and initial clinic visit was 3 days (Range: 0–9 days). Four pH1N1-positive patients had underlying medical conditions: Out of 16 patients who were tested for HIV, 2 were HIV-positive. Two patients had asthma (Table 1) and were the only enrolled patients who received oseltamivir treatment. None of the enrolled patients required hospitalization.

**Table 1:** Demographic characteristics of 285 patients with acute respiratory illness who presented to Kibera Clinic, Nairobi, Kenya and had specimens collected, Oct–Nov 2009.

Characteristics	rRT-PCR Results	
Age in years	pH1N1-positiveN (%)	pH1N1-negativeN (%)
<5	43 (40)	90(62)
5–14	41 (39)	32(22)
≥15	22 (21)	23(16)
**Gender**		
Male	46(43)	71(49)
Female	60(57)	74(51)
**Co-existing medical conditions**		
HIV	2(13)*	3(14)
Asthma	2 (2)	0
Treated with oseltamivir	2(2)	N/A

*16 people were tested for HIV.

### Pandemic H1N1 virus detection

**Figure 2:**
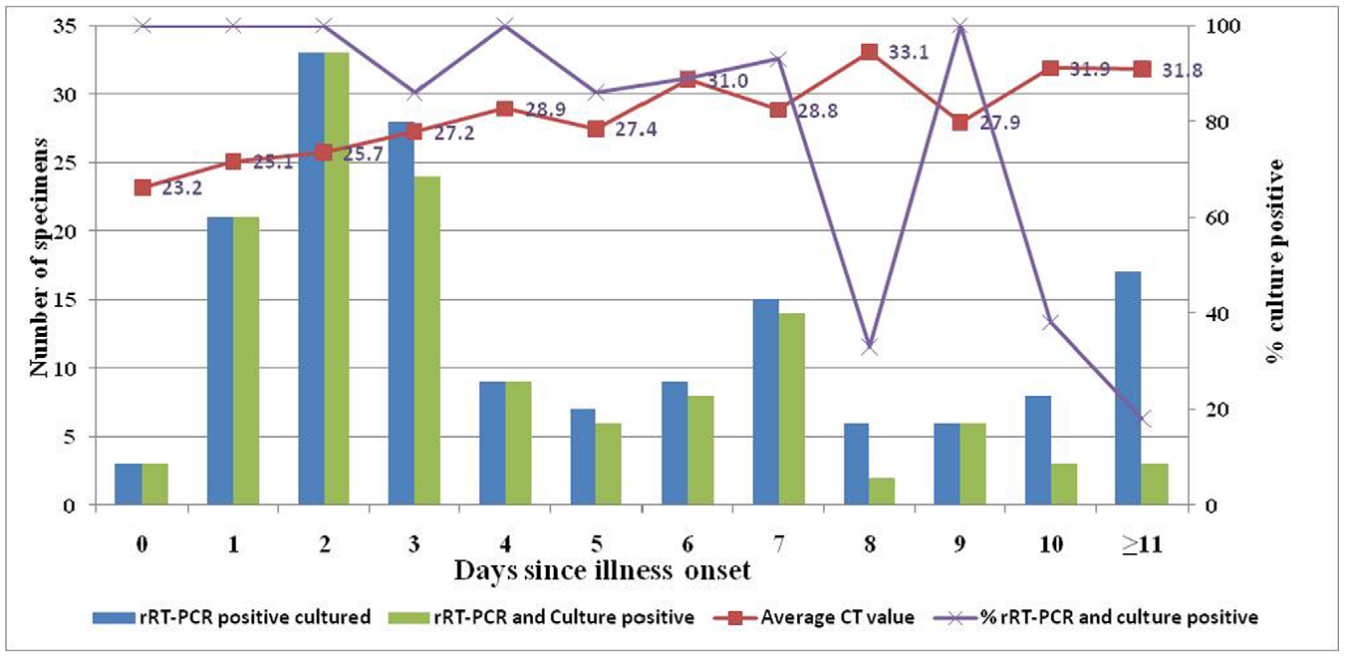
Correlation between rRT-PCR-positive results, cell culture results, and days of illness.

From the 106 patients enrolled to the study, 449 specimens were collected including the initial specimen from which pH1N1 was detected; 200 (44%) were positive for pH1N1 by rRT-PCR. Virus isolation was attempted in 262 of the 449 (58%) specimens collected. The specimens cultured included the 106 initial rRT-PCR-positive specimens, 56 final rRT-PCR-positive specimens (fifty patients had only one rRT-PCR-positive specimen), and100 first rRT-PCR-negative specimens from the enrolled patients. No rRT-PCR-negative specimens were obtained from 6 of the patients. Of the 162 rRT-PCR-positive specimens, pH1N1 virus was isolated from 132 (81%) specimens. Of rRT-PCR-positive specimens taken on day 0–3 after illness onset, 81/85 (95%) were culture-positive, as were 37/40 (93%) taken on day 4–7, 11/20 (55%) taken on day 8–10, and 3/17 (18%) taken ≥11 days after illness onset (Figure 2). Viral isolation was successful in 100/106 (94%) of the rRT-PCR positive specimens collected at the initial clinic visit. Viral isolation was equally successful in samples with C_T_ values<25 and those with C_T_ values 25–30 (92% vs. 84% p = 0.25), but more successful for samples with C_T_ values<25 compared to those with C_T_ values 30–39 (92% vs. 63%, p = 0.009). Real time RT-PCR-positive specimens collected from patients on day 0–3 after illness onset were 7.5 times more likely to be culture-positive than rRT-PCR-positive specimens collected on day 4–7 (CI: 1.4–38.9; P<0.012), 17.6 times more likely to be culture-positive than rRT-PCR-positive specimens collected on day 8–10 (CI: 3.2–96.1; p<0.001), and 142.9 times more likely to be culture-positive than specimens collected from patients ≥11 days after illness onset (CI: 2.4–833.3; P<0.001). Of the 100 rRT-PCR negative specimens, 6(6.4%) were culture-positive.

**Figure 3:**
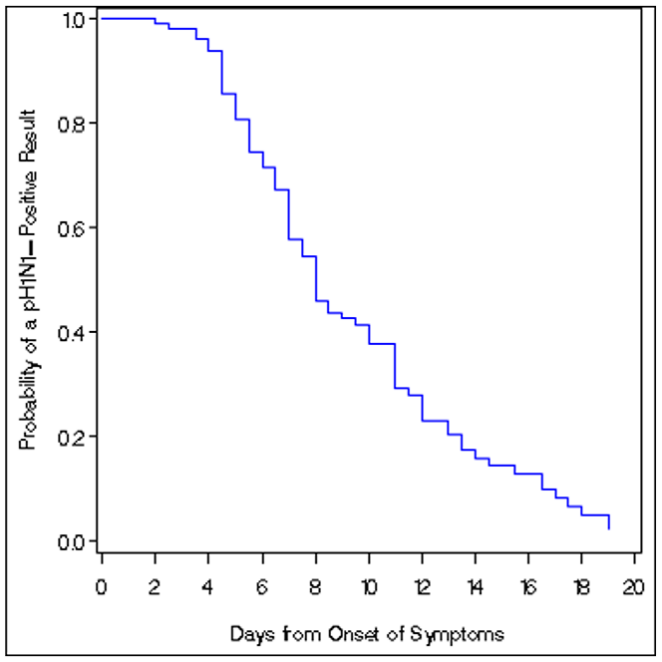
Kaplan Meier plot showing the probability of rRT-PCR-positive pH1N1 test result by day after symptom onset.

The median number of days pH1N1 virus RNA was detectable in patients specimens 8 days (95% CI: 7–10 days) after symptom onset. There was no statistically significant difference in the duration of pH1N1 virus detection between children <5 years old and those 5 to 14 years or persons ≥15 years. There was no difference in the median duration of pH1N1 virus detection between males and females. In the majority (58%) of patients, pH1N1 virus RNA was detected for ≥7 days, and in 16% of patients for ≥14 days (Figure 3). In general, the mean C_T_ value increased with time. One of the HIV-positive patients who was on antiretroviral therapy and had a CD4 count of 17cells/mm^3^ had detectable pH1N1 virus RNA for 4 days after symptom onset. The other HIV-positive patient, who was not on antiretroviral treatment and whose CD4 count was unknown, had detectable pH1N1 RNA for 16 days after symptom onset. These two patients completed the study with two consecutive pH1N1-negative specimens.

### Association between pH1N1 virus detection and clinical symptoms

There were 200 patient visits that were associated with pH1N1-positive rRT-PCR results. We were able to link laboratory results with clinic visit data on symptoms for 186/200 patients. From the linkable patient visits, there were 69/186 pH1N1-positive rRT-PCR results from patients <5 years old and 117/186 from patients ≥5 years old. The most common signs and symptoms in the pH1N1-positive patients <5 years old were cough [52/69 (75%)], runny nose [47/69 (68%)], reported fever [44/69 (64%)], and temperature ≥38°C [40/69 (58%)]. The most common signs and symptoms in the patients ≥5 years old were cough [83/117 (71%)], temperature ≥38°C [63/117 (54%)], reported fever [61/117 (52%)], and runny nose [60/117 (51%)] (Table 2).

**Table 2:** Signs and Symptoms associated with pH1N1 rRT-PCR-positive test results.

	Patients <5 yrs					Patients ≥5 yrs				
		Days after symptom onset				Days after symptom onset				
Symptom	*N	0–3	4–7	8–11	12–20	*N	0–3	4–7	8–11	12–20
Total Patient visits	69	36	15	14	4	117	50	29	19	19
Reported Fever (%)	44(64)	35(97)	7(47)	2(14)	0(0)	61(52)	45(90)	16(55)	0(0)	0(0)
Temp (≥38) (%)	40(58)	34(94)	6(40)	0(0)	0(0)	63(54)	49(98)	14(48)	0(0)	0(0)
Cough (%)	52(75)	33(92)	12(80)	5(36)	1(25)	83(71)	45(90)	23(79)	9(47)	6(32)
Sneezing (%)	24(35)	19(53)	4(27)	1(7)	0(0)	31(18)	20(40)	9(31)	2(11)	0(0)
Runny nose (%)	47(68)	31(86)	8(53)	6(43)	2(50)	60(51)	32(64)	17(59)	7(37)	4(22)
Vomiting (%)	17(25)	13(36)	3(20)	1(7)	0(0)	9(8)	6(12)	3(10)	0(0)	0(0)
Diarrhea (%)	8(12)	6(17)	2(13)	2(14)	0(0)	5(4)	2(4)	1(3)	1(5)	1(5)
Chest pain (%)	0(0)	0(0)	0(0)	0(0)	0(0)	23(20)	13(26)	7(24)	3(16)	0(0)
Difficulty Breathing (%)	6(9)	4(11)	2(13)	2(14)	0(0)	7(6)	4(8)	1(3)	2(11)	0(0)
Ear problem (%)*						6(5)	5(10)	0(0)	1(5)	0(0)
Sore throat (%)*						34(29)	22(44)	8(28)	3(16)	1(5)
Headache*						48(41)	30(60)	15(52)	2(11)	1(5)
Chills*						22(19)	14(28)	7(24)	1(5)	0(0)
Joint pain*						15(13)	8(16)	6(21)	1(5)	0(0)
Muscle pain*						10(9)	4(8)	5(17)	1(5)	0(00
Abdominal pain*						8(7)	5(10)	2(7)	1(5)	0(0)

*This information was not obtained from patients <5 years old.

Eighteen (17%) pH1N1 enrolled patients who were initially symptomatic and pH1N1-positive continued to be rRT-PCR-positive after all of their signs and symptoms had resolved for a median of 9 days (interquartile range 7–10 days) after all signs and symptoms had resolved. Viable pH1N1 virus was isolated from specimens obtained from 7/18(39%) of these patients.

## Discussion

This study describes the duration of pH1N1 virus RNA detection, the correlation between rRT-PCR-positive results and virus isolation, and clinical symptoms associated with pH1N1 virus detection in patients living in a densely populated community with low socioeconomic status in sub-Saharan Africa. Pandemic H1N1 RNA was detected from respiratory specimens by rRT-PCR for a median duration of 8 days but up to 17 days after symptoms onset. This duration of virus RNA detection is similar to the median of 6 and 8 days reported in studies from China and Hong Kong, respectively [7], [10]. In our study, we did not find any differences in the duration of pH1N1 RNA detection among various age groups or between males and females. These results were similar to those reported by a study in Hong Kong, which reported no correlation between influenza viral load and age [17], and one from Canada which showed no differences in shedding between children and adults [8]. In contrast, studies in Hong Kong and China found that younger age and male gender were risk factors for prolonged pH1N1 virus detection [7], [10]. Our study population, was mostly children (79% of the patients were <14 years old), thus associations between age and duration of pH1N1 RNA virus detection were difficult to assess. In addition, our study population included outpatients only, the China and Hong Kong studies mentioned above included hospitalized patients [7], [10].

Cough, fever, and runny nose were the most common clinical symptoms associated with pH1N1 virus RNA detection. This is similar to studies carried out in Korea [18] and Hong Kong [10]. While sore throat was commonly associated with pH1N1 patients in other studies [1], [10], [19], in our study, only 29% of the pH1N1-positive patient visits from patients ≥5 years old were associated with this symptom. In our study, a substantial proportion of patients continued to shed the virus after respiratory symptoms had resolved. Viable pH1N1 virus, which was potentially infectious, was isolated from one-third of specimens obtained from recovered patients. Therefore, some pH1N1 patients who were no longer symptomatic may still be shedding viable pH1N1 virus. In addition, half of rRT-PCR-positive samples from patients who were on day 8 to day 10 after symptom onset were culture-positive. In 2009, in the early stages of the H1N1 pandemic, WHO recommended that pH1N1 patients remain isolated for 7 days or until symptoms resolved [20]. While these guidelines may be appropriate for the community, in a healthcare setting, where the goal is to prevent as much spread as possible, other measures need to be considered [21].

Of the 2 HIV-positive patients in the study, the patient who was not on antiretroviral therapy (ART) had detectable pH1N1 virus RNA for 16 days, much longer than the median duration for the study population. This is slightly longer than was shown in a study of HIV-positive school-aged children carried out in Germany in which the median time from symptoms onset to first negative rRT-PCR result was 9 days (Range, 5–14 days) and cultures become negative after 6 days (Range 3–11 days) [22]. Other studies have shown that immunosuppressed individuals may shed influenza virus longer than the general population [2], [9], [19]. In a case report from the United States of America, 2 cancer patients who were severely immunosuppressed were shown to continue having detectable pH1N1 virus RNA for 5 and 6 weeks after initial diagnosis [9]. In previous studies, severely immunocompromised patients have been shown to shed seasonal influenza for weeks to months [2]. The issue of prolonged shedding in immunocompromised patients is especially relevant in sub-Saharan Africa, where over 22 million people are infected with HIV and only 30% are on ART [23], [24]. In Kenya, the HIV prevalence in persons aged 15–64 is 7.1% [25], and in the study site, HIV prevalence is 14% (KEMRI/CDC-K, unpublished data). In our study, we identified 2 HIV-positive patients among 16 tested, one of whom had prolonged shedding. More research should be conducted to better understand the extent of pH1N1 viral shedding in untreated HIV-infected patients and those on ART therapy.

PCR, which detects viral nucleic acid instead of infectious viral particles, is more sensitive than virus culture in detecting influenza [26]. However, because it does not detect viable whole virus, people who have respiratory specimens that are rRT-PCR-positive may not harbor a sufficient amount of viable virus to infect other people. The relationship between virus titers and influenza transmissibility is not known. However, serial interval studies suggest that most seasonal influenza household transmission occurs in the first 3 days after the index case's illness onset [21]. In our study, we successfully isolated pH1N1 virus from 95% (81/85) of rRT-PCR-positive specimens taken from day 0–3 after symptom onset, but only 18% (3/17) rRT-PCR-positive specimens taken ≥11 days after symptom onset were culture-positive. These findings suggest that if a patient has a respiratory specimen taken early in the course of illness that is positive for pH1N1 by rRT-PCR, that patient is likely shedding live virus. In contrast, a rRT-PCR-positive result from a sample taken later in a patient's course of illness may not mean that the patient is still shedding live virus.

Our study had some limitations. First, our study population was mainly comprised of persons <14 years, and the oldest patient was 41 years old. Thus, we were not able to evaluate viral shedding patterns in elderly individuals. Second, since few people in our study had known underlying medical conditions, we were not able to determine the impact of co-morbidities, including HIV infection, on duration of pH1N1 viral shedding. Some patients whose HIV status was unknown may have been HIV-positive; this could explain the slightly longer duration of rRT-PCR positive pH1N1 results in our study compared to other studies. Third, there was an overlap between the patients who presented with ILI and SARI, thus it was difficult to analyze data for each of the syndromes separately. Finally, 20% of patients enrolled in the study did not fully complete the study. Therefore, we have little information on these patients regarding shedding. We used right-censoring in our analysis for these patients. These results were comparable to those obtained from the 85 patients who completed the study.

In this study, we show that pH1N1 shedding patterns in an impoverished, densely populated urban community in Nairobi, Kenya, are similar to those described in studies in more affluent countries in temperate and subtropical areas of the world. Because of unique co-morbidities in sub-Saharan Africa compared to other areas of the world [27], more research is needed to characterize shedding dynamics and impact on disease transmission for pH1N1 infection in African communities.
